# Sodium quercetin-8-sulfonate trihydrate

**DOI:** 10.1107/S1600536810029570

**Published:** 2010-07-31

**Authors:** Xian Zhang, Yueqing Li, Pingping Chen, Tianjiao Han, Weijie Zhao

**Affiliations:** a School of Pharmaceutical Science and Technology, Dalian Unversity of Technology, PO Box 90, Zhongshan Road 158, Dalian 116012, People’s Republic of China

## Abstract

The organic anion of the title compound, {[Na(C_15_H_9_O_10_S)(H_2_O)_2_]·H_2_O}_*n*_ {systematic name: poly[[diaqua­[μ-2-(3,4-dihy­droxy­phen­yl)-3,5,7-trihy­droxy-4-oxo-4*H*-chromene-8-sulfon­ato]­sodium] monohydrate]}, has a nearly planar structure. The Na atom is six-coordinated by O atoms, two from water mol­ecules and four from the anion. The dihedral angle between the ring systems in the anion is 10.1 (1)°. Intra­molecular O—H⋯S and O—H⋯O inter­actions occur. In the crystal structure, an extensive network of classical inter­molecular O—H⋯S and O—H⋯O hydrogen bonds forms layers along the *c* axis.

## Related literature

The title compound is of inter­est for its potential anti-inflammatory and anti­viral properties. For the synthesis and structures of analogues of the title compound, see: Kopacz *et al.* (1978 ▶, 1983 ▶); Cheng (2006 ▶); Wang (2007 ▶); Liu *et al.* (2009 ▶). For the anti-HIV properties of flavonoids and their derivatives, see: Kashiwada *et al.* (2005 ▶); Lameira *et al.* (2006 ▶); Reutrakul *et al.* (2007 ▶); Li *et al.* (2010 ▶).
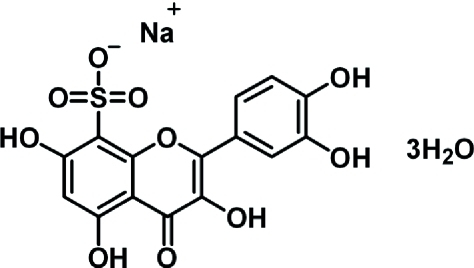


## Experimental

### 

#### Crystal data

[Na(C_15_H_9_O_10_S)(H_2_O)_2_]·H_2_O
*M* *_r_* = 458.33
Triclinic, 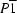

*a* = 7.595 (3) Å
*b* = 10.157 (3) Å
*c* = 12.183 (4) Å
α = 76.576 (4)°
β = 81.031 (4)°
γ = 77.385 (3)°
*V* = 886.6 (5) Å^3^
*Z* = 2
Mo *K*α radiation
μ = 0.28 mm^−1^
*T* = 295 K
0.60 × 0.31 × 0.24 mm

#### Data collection

Bruker SMART APEX CCD diffractometer
4109 measured reflections
2994 independent reflections
2691 reflections with *I* > 2σ(*I*)
*R*_int_ = 0.013

#### Refinement

*R*[*F* ^2^ > 2σ(*F* ^2^)] = 0.034
*wR*(*F* ^2^) = 0.101
*S* = 1.01
2994 reflections
289 parameters
H atoms treated by a mixture of independent and constrained refinement
Δρ_max_ = 0.24 e Å^−3^
Δρ_min_ = −0.41 e Å^−3^

### 

Data collection: *APEX2* (Bruker, 2005 ▶); cell refinement: *SAINT-Plus* (Bruker, 2001 ▶); data reduction: *SAINT-Plus*; program(s) used to solve structure: *SHELXS97* (Sheldrick, 2008 ▶); program(s) used to refine structure: *SHELXL97* (Sheldrick, 2008 ▶); molecular graphics: *SHELXTL* (Sheldrick, 2008 ▶); software used to prepare material for publication: *SHELXTL*.

## Supplementary Material

Crystal structure: contains datablocks I, global. DOI: 10.1107/S1600536810029570/rk2218sup1.cif

Structure factors: contains datablocks I. DOI: 10.1107/S1600536810029570/rk2218Isup2.hkl

Additional supplementary materials:  crystallographic information; 3D view; checkCIF report

## Figures and Tables

**Table 1:** Selected bond lengths (Å)

Na1—O2^i^	2.3517 (16)
Na1—O6^ii^	2.3784 (16)
Na1—O1^ii^	2.3919 (17)
Na1—O13	2.4070 (18)
Na1—O12	2.555 (2)
Na1—O9	2.4074 (15)

Symmetry codes: (i) 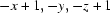; (ii) 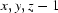.

**Table 2:** Hydrogen-bond geometry (Å, °)

*D*—H⋯*A*	*D*—H	H⋯*A*	*D*⋯*A*	*D*—H⋯*A*
O1—H1*A*⋯O13^iii^	0.82	1.88	2.677 (2)	164
O4—H4*A*⋯O2^i^	0.82	1.97	2.786 (2)	170
O4—H4*A*⋯S1^i^	0.82	2.97	3.7139 (18)	152
O5—H5*B*⋯O9	0.82	1.89	2.619 (2)	148
O6—H6*A*⋯O12^i^	0.82	1.87	2.688 (2)	177
O8—H8*B*⋯O3	0.82	1.85	2.596 (2)	152
O8—H8*B*⋯S1	0.82	2.65	3.1344 (16)	120
O13—H13*A*⋯O11^iv^	0.85 (3)	1.95 (3)	2.799 (3)	174 (3)
O13—H13*B*⋯O7^v^	0.79 (3)	2.22 (3)	2.983 (2)	161 (3)
O13—H13*B*⋯O9	0.79 (3)	2.64 (3)	3.017 (2)	111 (2)
O13—H13*B*⋯S1^v^	0.79 (3)	3.02 (3)	3.7333 (18)	152 (3)
O12—H12*A*⋯O11^vi^	0.83 (3)	2.07 (3)	2.850 (3)	155 (3)
O12—H12*B*⋯O7^i^	0.90 (3)	1.87 (3)	2.767 (2)	172 (3)
O12—H12*B*⋯S1^i^	0.90 (3)	2.74 (3)	3.494 (2)	142 (2)
O11—H11*A*⋯O5^vii^	0.86 (4)	2.10 (4)	2.914 (3)	158 (3)
O11—H11*B*⋯O3	0.87 (4)	1.99 (4)	2.832 (2)	163 (3)

Symmetry codes: (i) 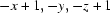; (iii) 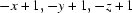; (iv) 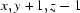; (v) 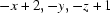; (vi) 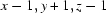; (vii) 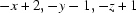.

**Fig. 1.**
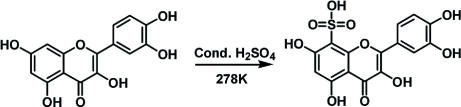
The synthetic route for title compound.

**Fig. 2.**
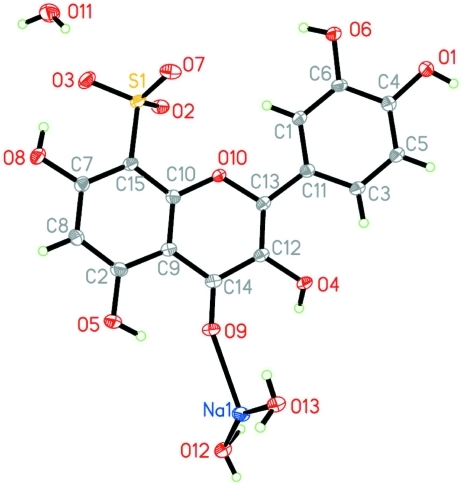
The part of molecular structure of title compound, showing the atom numbering scheme. Displacement ellipsoids are drawn at 30% probability level. H atoms are presented as a small spheres of arbitrary radius.

**Fig. 3.**
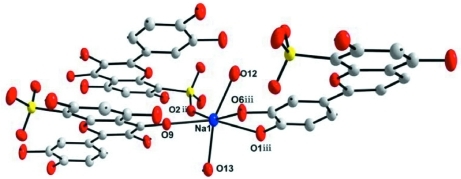
The oxygen environment of sodium cation. H atoms are omitted for clarity. Symmetry codes see in table of geometric parameters.

**Fig. 4.**
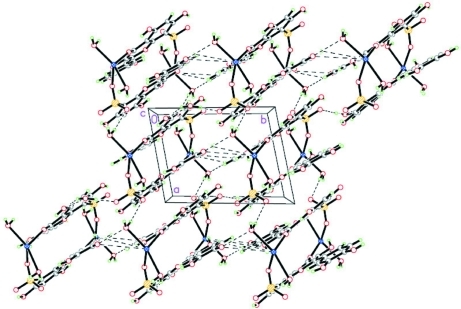
A view of the packing of title compound. Dashed lines indicate O—H···O hydrogen bonds.

## supplementary crystallographic information

### Comment

The flavonoids and their derivants have been investigated for a long time for their notable antiviral activity especially against *HIV*-1 (Kashiwada *et al.*, 2005; Lameira *et al.*, 2006; Reutrakul *et al.*, 2007). A great many of substituents have been applied to modify the structures to develop their solubility in water such as sulfonic group (Cheng, 2006; Kopacz, *et al.*, 1978; Kopacz *et al.*, 1983; Liu *et al.*, 2009; Wang, 2007). The title compound is an excellent antagonist of *Vif* which has been found to be a novel hit of *HIV*–1 (Li *et al.*, 2010). The crystal structure of the title compound may be helpful to the understanding of quantitative structure–activity relationship.

Quercetin–8–sulfonate sodium is synthesized from quercetin *via* sulfonation (Fig.1). The asymmetric unit of the title compound contains a quercetin–8–sulfonic anion, a sodium cation and three molecules of water (Fig.2). The anion structure is nearly coplanar.

In the crystal structure, sodium cation form six contacts 2.392 (2)–2.555 (2)Å (Fig.3) with oxygen atoms: two water atoms and four with other atoms of anion.

The hydrogen bonds assist crystal packing in layers along the *c* axis, (Table 1, Fig. 4).

### Refinement

The hydrogen atoms based on C were refined as riding on their parent atoms with C—H = 0.93Å and *U*_iso_(H) = 1.2*U*_eq_(C) for aromatic H; H atoms of hydroxy groups with O—H = 0.82Å and *U*_iso_(H) = 1.5*U*_eq_(O). For H atoms of water molecules, positions were refined freely and *U*_iso_(H) = 1.5*U*_eq_(O).

### Crystal data

**Table:**

[Na(C_15_H_9_O_10_S)(H_2_O)_2_]·H_2_O	*Z* = 2
*M**_r_* = 458.33	*F*(000) = 472
Triclinic, *P*1	*D*_x_ = 1.717 Mg m^−^^3^
Hall symbol: -P 1	Mo *K*α radiation, λ = 0.71073 Å
*a* = 7.595 (3) Å	Cell parameters from 3467 reflections
*b* = 10.157 (3) Å	θ = 2.3–26.2°
*c* = 12.183 (4) Å	µ = 0.28 mm^−^^1^
α = 76.576 (4)°	*T* = 295 K
β = 81.031 (4)°	Needle, pale yellow
γ = 77.385 (3)°	0.60 × 0.31 × 0.24 mm
*V* = 886.6 (5) Å^3^	

### Data collection

**Table:**

Bruker SMART APEX CCD diffractometer	2691 reflections with *I* > 2σ(*I*)
Radiation source: fine–focus sealed tube	*R*_int_ = 0.013
graphite	θ_max_ = 25.0°, θ_min_ = 2.4°
φ– and ω–scans	*h* = −9→8
4109 measured reflections	*k* = −12→10
2994 independent reflections	*l* = −14→9

### Refinement

**Table:**

Refinement on *F*^2^	Primary atom site location: structure-invariant direct methods
Least-squares matrix: full	Secondary atom site location: difference Fourier map
*R*[*F*^2^ > 2σ(*F*^2^)] = 0.034	Hydrogen site location: inferred from neighbouring sites
*wR*(*F*^2^) = 0.101	H atoms treated by a mixture of independent and constrained refinement
*S* = 1.01	*w* = 1/[σ^2^(*F*_o_^2^) + (0.0667*P*)^2^ + 0.3015*P*] where *P* = (*F*_o_^2^ + 2*F*_c_^2^)/3
2994 reflections	(Δ/σ)_max_ < 0.001
289 parameters	Δρ_max_ = 0.24 e Å^−^^3^
0 restraints	Δρ_min_ = −0.41 e Å^−^^3^

### Special details

**Table:**

Experimental. The synthesis of title compound is shown in Fig. 1. The crude product was recrystallized by CH_3_OH/H_2_O = 3/1 in the yield of 60%. However, the single crystals were obtained in 0.9% NaCl aqueous solution at a concentration of 0.3 mg ml^-1^.
Geometry. All s.u.'s (except the s.u. in the dihedral angle between two l.s. planes) are estimated using the full covariance matrix. The cell s.u.'s are taken into account individually in the estimation of s.u.'s in distances, angles and torsion angles; correlations between s.u.'s in cell parameters are only used when they are defined by crystal symmetry. An approximate (isotropic) treatment of cell s.u.'s is used for estimating s.u.'s involving l.s. planes.
Refinement. Refinement of *F*^2^ against ALL reflections. The weighted *R*–factor *wR* and goodness of fit *S* are based on *F*^2^, conventional *R*–factors *R* are based on *F*, with *F* set to zero for negative *F*^2^. The threshold expression of *F*^2^ > σ(*F*^2^) is used only for calculating *R*–factors(gt) *etc*. and is not relevant to the choice of reflections for refinement. *R*–factors based on *F*^2^ are statistically about twice as large as those based on *F*, and *R*–factors based on ALL data will be even larger.

### Fractional atomic coordinates and isotropic or equivalent isotropic displacement parameters (Å^2^)

**Table:**

	*x*	*y*	*z*	*U*_iso_*/*U*_eq_	
S1	0.91509 (6)	−0.29304 (4)	0.71144 (4)	0.02554 (15)	
O1	0.4645 (2)	0.37543 (14)	0.91755 (12)	0.0410 (4)	
H1A	0.4085	0.4545	0.8995	0.062*	
O2	0.72475 (18)	−0.29014 (14)	0.75277 (11)	0.0316 (3)	
O3	1.0212 (2)	−0.43287 (15)	0.73041 (12)	0.0423 (4)	
O4	0.5196 (2)	0.27317 (13)	0.40069 (11)	0.0347 (3)	
H4A	0.4517	0.2682	0.3563	0.052*	
O5	0.9040 (2)	−0.11772 (16)	0.21432 (11)	0.0427 (4)	
H5B	0.8299	−0.0458	0.2001	0.064*	
O6	0.6438 (2)	0.11901 (14)	0.94873 (11)	0.0409 (4)	
H6A	0.6838	0.0365	0.9526	0.061*	
O7	0.99190 (19)	−0.20151 (15)	0.75754 (12)	0.0373 (3)	
O8	1.14625 (18)	−0.43644 (14)	0.52006 (12)	0.0357 (3)	
H8B	1.1330	−0.4599	0.5895	0.054*	
O9	0.66041 (18)	0.09603 (13)	0.25617 (11)	0.0308 (3)	
O10	0.74224 (17)	−0.02632 (12)	0.58747 (10)	0.0260 (3)	
C1	0.6350 (3)	0.11097 (19)	0.75271 (16)	0.0276 (4)	
H1B	0.6968	0.0200	0.7645	0.033*	
C2	0.9124 (3)	−0.1552 (2)	0.32742 (16)	0.0293 (4)	
C3	0.4925 (3)	0.31432 (19)	0.62889 (16)	0.0297 (4)	
H3B	0.4576	0.3606	0.5580	0.036*	
C4	0.5007 (3)	0.31472 (19)	0.82552 (16)	0.0296 (4)	
C5	0.4522 (3)	0.38082 (19)	0.71898 (17)	0.0317 (4)	
H5A	0.3912	0.4720	0.7077	0.038*	
C6	0.5936 (3)	0.17823 (19)	0.84191 (16)	0.0280 (4)	
C7	1.0321 (2)	−0.31769 (18)	0.48751 (16)	0.0267 (4)	
C8	1.0238 (3)	−0.2759 (2)	0.37051 (17)	0.0314 (4)	
H8A	1.0939	−0.3301	0.3217	0.038*	
C9	0.8119 (2)	−0.06762 (18)	0.39941 (15)	0.0244 (4)	
C10	0.8272 (2)	−0.10881 (18)	0.51493 (15)	0.0239 (4)	
C11	0.5857 (2)	0.17729 (18)	0.64382 (15)	0.0238 (4)	
C12	0.6164 (2)	0.14599 (18)	0.43889 (15)	0.0250 (4)	
C13	0.6439 (2)	0.10360 (18)	0.55063 (15)	0.0239 (4)	
C14	0.6942 (2)	0.05993 (18)	0.35794 (15)	0.0241 (4)	
C15	0.9291 (2)	−0.23665 (18)	0.56233 (15)	0.0245 (4)	
Na1	0.49543 (10)	0.22781 (8)	0.10033 (6)	0.0340 (2)	
O13	0.7175 (2)	0.36463 (15)	0.09986 (14)	0.0366 (3)	
H13A	0.790 (4)	0.362 (3)	0.039 (2)	0.055*	
H13B	0.776 (4)	0.325 (3)	0.150 (2)	0.055*	
O12	0.2274 (2)	0.15022 (17)	0.04739 (15)	0.0459 (4)	
H12A	0.182 (4)	0.209 (3)	−0.006 (3)	0.069*	
H12B	0.151 (4)	0.161 (3)	0.110 (3)	0.069*	
O11	0.9733 (3)	−0.6600 (2)	0.91012 (15)	0.0564 (5)	
H11A	0.982 (5)	−0.731 (4)	0.882 (3)	0.085*	
H11B	0.986 (5)	−0.582 (4)	0.866 (3)	0.085*	

### Atomic displacement parameters (Å^2^)

**Table:**

	*U*^11^	*U*^22^	*U*^33^	*U*^12^	*U*^13^	*U*^23^
S1	0.0235 (3)	0.0283 (3)	0.0212 (3)	−0.00011 (18)	−0.00574 (18)	−0.00029 (18)
O1	0.0618 (10)	0.0307 (7)	0.0262 (8)	0.0075 (7)	−0.0099 (7)	−0.0089 (6)
O2	0.0257 (7)	0.0430 (8)	0.0247 (7)	−0.0058 (6)	−0.0031 (5)	−0.0045 (6)
O3	0.0463 (9)	0.0363 (8)	0.0297 (8)	0.0110 (7)	−0.0030 (6)	0.0039 (6)
O4	0.0476 (9)	0.0261 (7)	0.0292 (7)	0.0043 (6)	−0.0207 (6)	−0.0031 (6)
O5	0.0553 (10)	0.0449 (8)	0.0197 (7)	0.0086 (7)	−0.0050 (6)	−0.0063 (6)
O6	0.0618 (10)	0.0324 (7)	0.0219 (7)	0.0103 (7)	−0.0124 (7)	−0.0051 (6)
O7	0.0377 (8)	0.0490 (9)	0.0286 (7)	−0.0148 (7)	−0.0095 (6)	−0.0045 (6)
O8	0.0338 (8)	0.0335 (7)	0.0316 (8)	0.0091 (6)	−0.0045 (6)	−0.0042 (6)
O9	0.0346 (7)	0.0344 (7)	0.0214 (7)	−0.0050 (6)	−0.0085 (6)	0.0002 (6)
O10	0.0303 (7)	0.0240 (6)	0.0201 (6)	0.0031 (5)	−0.0057 (5)	−0.0027 (5)
C1	0.0314 (10)	0.0235 (9)	0.0250 (10)	−0.0009 (7)	−0.0059 (8)	−0.0016 (7)
C2	0.0290 (10)	0.0354 (10)	0.0215 (9)	−0.0047 (8)	−0.0025 (7)	−0.0035 (8)
C3	0.0321 (10)	0.0292 (10)	0.0253 (10)	0.0012 (8)	−0.0099 (8)	−0.0028 (8)
C4	0.0311 (10)	0.0292 (10)	0.0280 (10)	−0.0023 (8)	−0.0045 (8)	−0.0073 (8)
C5	0.0347 (11)	0.0266 (9)	0.0299 (10)	0.0041 (8)	−0.0083 (8)	−0.0041 (8)
C6	0.0317 (10)	0.0293 (10)	0.0205 (9)	−0.0021 (8)	−0.0070 (7)	−0.0008 (8)
C7	0.0220 (9)	0.0277 (9)	0.0286 (10)	−0.0020 (7)	−0.0034 (7)	−0.0040 (8)
C8	0.0304 (10)	0.0341 (10)	0.0270 (10)	−0.0008 (8)	0.0010 (8)	−0.0088 (8)
C9	0.0232 (9)	0.0268 (9)	0.0225 (9)	−0.0058 (7)	−0.0040 (7)	−0.0015 (7)
C10	0.0211 (9)	0.0268 (9)	0.0230 (9)	−0.0041 (7)	−0.0021 (7)	−0.0046 (7)
C11	0.0226 (9)	0.0245 (9)	0.0244 (9)	−0.0048 (7)	−0.0051 (7)	−0.0031 (7)
C12	0.0241 (9)	0.0244 (9)	0.0258 (9)	−0.0048 (7)	−0.0074 (7)	−0.0005 (7)
C13	0.0213 (9)	0.0222 (8)	0.0266 (10)	−0.0025 (7)	−0.0064 (7)	−0.0007 (7)
C14	0.0219 (9)	0.0285 (9)	0.0219 (9)	−0.0092 (7)	−0.0044 (7)	0.0004 (7)
C15	0.0223 (9)	0.0269 (9)	0.0224 (9)	−0.0029 (7)	−0.0040 (7)	−0.0023 (7)
Na1	0.0363 (4)	0.0398 (4)	0.0239 (4)	−0.0026 (3)	−0.0038 (3)	−0.0064 (3)
O13	0.0419 (9)	0.0356 (8)	0.0295 (8)	−0.0001 (6)	−0.0124 (6)	−0.0025 (6)
O12	0.0565 (10)	0.0388 (8)	0.0330 (9)	0.0063 (7)	−0.0072 (8)	−0.0014 (7)
O11	0.0839 (14)	0.0463 (10)	0.0333 (9)	−0.0074 (9)	−0.0062 (9)	−0.0016 (8)

### Geometric parameters (Å, °)

**Table:**

S1—O7	1.4498 (15)	C3—C11	1.401 (3)
S1—O2	1.4510 (15)	C3—H3B	0.9300
S1—O3	1.4581 (15)	C4—C5	1.381 (3)
S1—C15	1.7671 (19)	C4—C6	1.396 (3)
O1—C4	1.365 (2)	C5—H5A	0.9300
O1—Na1^i^	2.3919 (17)	C7—C8	1.397 (3)
O1—H1A	0.8200	C7—C15	1.402 (3)
O2—Na1^ii^	2.3517 (16)	C8—H8A	0.9300
O4—C12	1.356 (2)	C9—C10	1.388 (3)
O4—H4A	0.8200	C9—C14	1.437 (3)
O5—C2	1.349 (2)	C10—C15	1.404 (2)
O5—H5B	0.8200	C11—C13	1.462 (3)
O6—C6	1.375 (2)	C12—C13	1.364 (3)
O6—Na1^i^	2.3784 (16)	C12—C14	1.442 (3)
O6—H6A	0.8200	Na1—O2^ii^	2.3517 (16)
O8—C7	1.339 (2)	Na1—O6^iii^	2.3784 (16)
O8—H8B	0.8200	Na1—O1^iii^	2.3919 (17)
O9—C14	1.259 (2)	Na1—O13	2.4070 (18)
O9—Na1	2.4074 (15)	Na1—O12	2.555 (2)
O10—C10	1.353 (2)	Na1—O9	2.4074 (15)
O10—C13	1.378 (2)	O13—H13A	0.85 (3)
C1—C6	1.375 (3)	O13—H13B	0.79 (3)
C1—C11	1.409 (3)	O12—H12A	0.83 (3)
C1—H1B	0.9300	O12—H12B	0.90 (3)
C2—C8	1.371 (3)	O11—H11A	0.86 (4)
C2—C9	1.414 (3)	O11—H11B	0.87 (4)
C3—C5	1.380 (3)		
			
O7—S1—O2	112.09 (8)	C10—C9—C14	119.29 (16)
O7—S1—O3	111.69 (9)	C2—C9—C14	122.76 (17)
O2—S1—O3	111.94 (9)	O10—C10—C9	120.78 (16)
O7—S1—C15	108.31 (8)	O10—C10—C15	116.87 (16)
O2—S1—C15	107.22 (8)	C9—C10—C15	122.35 (17)
O3—S1—C15	105.18 (8)	C3—C11—C1	117.98 (16)
C4—O1—Na1^i^	117.41 (12)	C3—C11—C13	123.15 (16)
C4—O1—H1A	109.5	C1—C11—C13	118.75 (16)
Na1^i^—O1—H1A	130.6	O4—C12—C13	120.25 (16)
S1—O2—Na1^ii^	141.71 (9)	O4—C12—C14	118.36 (15)
C12—O4—H4A	109.5	C13—C12—C14	121.35 (16)
C2—O5—H5B	109.5	C12—C13—O10	119.16 (16)
C6—O6—Na1^i^	117.44 (11)	C12—C13—C11	129.68 (16)
C6—O6—H6A	109.5	O10—C13—C11	111.15 (15)
Na1^i^—O6—H6A	126.9	O9—C14—C9	122.20 (17)
C7—O8—H8B	109.5	O9—C14—C12	121.20 (16)
C14—O9—Na1	155.71 (12)	C9—C14—C12	116.61 (16)
C10—O10—C13	122.23 (14)	C7—C15—C10	117.60 (17)
C6—C1—C11	121.25 (16)	C7—C15—S1	122.63 (14)
C6—C1—H1B	119.4	C10—C15—S1	119.70 (14)
C11—C1—H1B	119.4	O2^ii^—Na1—O6^iii^	160.25 (6)
O5—C2—C8	119.29 (17)	O2^ii^—Na1—O1^iii^	115.40 (6)
O5—C2—C9	119.81 (17)	O6^iii^—Na1—O1^iii^	67.01 (5)
C8—C2—C9	120.86 (17)	O2^ii^—Na1—O13	102.00 (6)
C5—C3—C11	120.22 (17)	O6^iii^—Na1—O13	97.74 (6)
C5—C3—H3B	119.9	O1^iii^—Na1—O13	81.25 (6)
C11—C3—H3B	119.9	O2^ii^—Na1—O9	82.95 (6)
O1—C4—C5	123.83 (17)	O6^iii^—Na1—O9	101.98 (6)
O1—C4—C6	116.98 (17)	O1^iii^—Na1—O9	154.60 (6)
C5—C4—C6	119.19 (17)	O13—Na1—O9	77.62 (6)
C3—C5—C4	121.35 (17)	O2^ii^—Na1—O12	81.05 (6)
C3—C5—H5A	119.3	O6^iii^—Na1—O12	80.12 (6)
C4—C5—H5A	119.3	O1^iii^—Na1—O12	80.40 (6)
C1—C6—O6	123.26 (16)	O13—Na1—O12	160.82 (6)
C1—C6—C4	120.01 (17)	O9—Na1—O12	121.53 (6)
O6—C6—C4	116.70 (16)	Na1—O13—H13A	107.5 (19)
O8—C7—C8	115.11 (16)	Na1—O13—H13B	106 (2)
O8—C7—C15	124.11 (17)	H13A—O13—H13B	106 (3)
C8—C7—C15	120.77 (17)	Na1—O12—H12A	109 (2)
C2—C8—C7	120.17 (17)	Na1—O12—H12B	98.1 (19)
C2—C8—H8A	119.9	H12A—O12—H12B	106 (3)
C7—C8—H8A	119.9	H11A—O11—H11B	120 (3)
C10—C9—C2	117.94 (16)		

Symmetry codes: (i) *x*, *y*, *z*+1; (ii) −*x*+1, −*y*, −*z*+1; (iii) *x*, *y*, *z*−1.

### Hydrogen-bond geometry (Å, °)

**Table:**

*D*—H···*A*	*D*—H	H···*A*	*D*···*A*	*D*—H···*A*
O1—H1A···O13^iv^	0.82	1.88	2.677 (2)	164
O4—H4A···O2^ii^	0.82	1.97	2.786 (2)	170
O4—H4A···S1^ii^	0.82	2.97	3.7139 (18)	152
O5—H5B···O9	0.82	1.89	2.619 (2)	148
O6—H6A···O12^ii^	0.82	1.87	2.688 (2)	177
O8—H8B···O3	0.82	1.85	2.596 (2)	152
O8—H8B···S1	0.82	2.65	3.1344 (16)	120
O13—H13A···O11^v^	0.85 (3)	1.95 (3)	2.799 (3)	174 (3)
O13—H13B···O7^vi^	0.79 (3)	2.22 (3)	2.983 (2)	161 (3)
O13—H13B···O9	0.79 (3)	2.64 (3)	3.017 (2)	111 (2)
O13—H13B···S1^vi^	0.79 (3)	3.02 (3)	3.7333 (18)	152 (3)
O12—H12A···O11^vii^	0.83 (3)	2.07 (3)	2.850 (3)	155 (3)
O12—H12B···O7^ii^	0.90 (3)	1.87 (3)	2.767 (2)	172 (3)
O12—H12B···S1^ii^	0.90 (3)	2.74 (3)	3.494 (2)	142 (2)
O11—H11A···O5^viii^	0.86 (4)	2.10 (4)	2.914 (3)	158 (3)
O11—H11B···O3	0.87 (4)	1.99 (4)	2.832 (2)	163 (3)

Symmetry codes: (iv) −*x*+1, −*y*+1, −*z*+1; (ii) −*x*+1, −*y*, −*z*+1; (v) *x*, *y*+1, *z*−1; (vi) −*x*+2, −*y*, −*z*+1; (vii) *x*−1, *y*+1, *z*−1; (viii) −*x*+2, −*y*−1, −*z*+1.
